# Gamma-Glutamyltransferase and Cancer Incidence: The Ohsaki Cohort Study

**DOI:** 10.2188/jea.JE20110071

**Published:** 2012-03-05

**Authors:** Toru Tsuboya, Shinichi Kuriyama, Masato Nagai, Atsushi Hozawa, Yumi Sugawara, Yasutake Tomata, Masako Kakizaki, Yoshikazu Nishino, Ichiro Tsuji

**Affiliations:** 1 Division of Epidemiology, Department of Public Health and Forensic Medicine, Tohoku University Graduate School of Medicine, Sendai, Japan; 2 Division of Molecular Epidemiology, Environment and Genome Research Center, Tohoku University Graduate School of Medicine, Sendai, Japan; 3 Department of Public Health, Yamagata University School of Medicine, Yamagata, Japan; 4 Division of Cancer Epidemiology and Prevention, Miyagi Cancer Center Research Institute, Natori, Japan

**Keywords:** gamma-glutamyltransferase, cancer incidence, population-based, prospective study, Ohsaki Study

## Abstract

**Background:**

Although experimental studies have shown that gamma-glutamyltransferase (GGT) has a role in tumor progression, epidemiologic evidence for a relationship between GGT and cancer incidence is limited. The present study investigated the association between GGT and cancer incidence and assessed the role of alcohol consumption in this association.

**Methods:**

We examined a cohort of 15 031 Japanese adults aged 40 to 79 years who attended a health checkup in 1995 and were free of cancer at that time. GGT was measured using the Szasz method. The participants were then followed from 1 January 1996 until 31 December 2005, and cancer incidence was recorded by using the Miyagi Regional Cancer Registry. Hazard ratios (HRs) and 95% confidence intervals (CIs) were computed for each quartile of GGT and compared. The lowest quartile (GGT <13.0 IU/ml) was used as the reference category.

**Results:**

We documented 1505 cancers. Among participants in the highest quartile (GGT ≥31.0 IU/ml), the multivariate HR for any cancer was 1.28 (95% CI, 1.08–1.53; *P* for trend, <0.001), the HR for colorectal cancer was significantly greater than unity, and the HRs for esophageal, pancreatic, and breast cancers were greater than unity but not significantly so. This positive trend was observed only in current drinkers.

**Conclusions:**

Our findings suggest that there is a positive relationship between GGT and cancer incidence only for alcohol-related cancers in current drinkers and that the positive association of GGT with cancer incidence largely reflects alcohol consumption.

## INTRODUCTION

In primary clinical settings, gamma-glutamyltransferase (GGT) is often measured to detect liver diseases such as hepatitis, fatty liver, and liver cancer. Experimental data indicate that GGT is critical in maintaining intracellular levels of glutathione (GSH), which protects cells against damage due to oxidation and free radicals.^1^^–^^5^ Thus, GGT is a sensitive and reliable marker of oxidative stress, a key factor in tumor progression.^6^^–^^9^ The effect of oxidative stress on carcinogenesis is not specific to liver cancer but rather is generalizable to all cancers.

To our knowledge, only 1 previous epidemiologic study examined the relationship between GGT elevation and cancer incidence.^10^^,^^11^ Data from the Vorarlberg Health Monitoring and Promotion Program (VHM&PP) in Austria showed a positive relationship between GGT and cancer incidence after adjustment for age, body mass index, smoking status, occupational status, and year of entry into the cohort. In that study, however, there was no information on alcohol consumption. Because alcohol consumption is related to both GGT level and the risk of some cancers, including cancer of the esophagus, liver, pancreas, colorectum, liver, and breast,^12^^–^^15^ its effect on the association between GGT and cancer incidence should be examined.

The present study investigated the association between GGT and cancer incidence and assessed the effect of alcohol consumption on this association. To our knowledge, this is the first prospective, population-based investigation of the association between GGT and cancer incidence, adjusted for alcohol consumption.

## METHODS

### Study cohort

We conducted this population-based cohort study by using data from the Ohsaki National Health Insurance (NHI) Cohort. The details of the Ohsaki National Health Insurance (NHI) Cohort have been previously published.^16^^–^^19^ In brief, between October and December 1994, we delivered a self-reported questionnaire to all individuals aged 40 to 79 years who were enrolled in the NHI and lived in the district covered by the Ohsaki Public Health Center, Miyagi Prefecture, in northeastern Japan. The Ohsaki Public Health Center is a local government agency that provides preventive health services for residents of 14 municipalities in northern Miyagi Prefecture. Of 54 996 eligible individuals, 52 029 (95%) responded. We started prospective collection of the NHI files on withdrawal history on 1 January 1995 to ascertain the dates of and reasons for withdrawal from the NHI. We excluded 776 participants who had withdrawn from the NHI before the baseline questionnaire survey. Thus, 51 253 participants (24 573 men and 26 680 women) were entered into the present study as our cohort participants. The ethics committee of the Tohoku University School of Medicine reviewed and approved the study protocol. The return of self-administered questionnaires signed by the participants was regarded as consent to take part in the study.

For the present analysis, we excluded 3170 participants (1571 men and 1599 women) who had received a diagnosis of cancer before the baseline survey was conducted, as determined by self-report or the Miyagi Prefectural Cancer Registry (described below), which resulted in a total of 48 083 participants, of whom 15 031 attended the annual health checkup in 1995. In Japan, the Health and Medical Service Law for the Aged requires all municipalities to provide annual health checkups for all residents aged 40 years or older. The checkup consists of an interview, measurement of weight, height, and blood pressure, a physical examination, and blood sampling. Nonfasting blood samples were obtained from all 15 031 participants. We combined these health checkup data with our original cohort. In the present study, data from the subcohort of 15 031 participants (6372 men and 8659 women) were used to investigate the association between GGT level and cancer incidence.

### Follow-up and ascertainment of cancer incidence

We followed the participants from 1 January 1996 to 31 December 2005. The end point was diagnosis of cancer, end of follow-up, death, emigration, or loss of NHI qualification, whichever occurred first. Using the NHI files on withdrawal history, we collected data on withdrawals from the NHI that occurred due to death, emigration, or loss of NHI qualification. We ascertained cancer incidence by computer linkage with the Miyagi Prefectural Cancer Registry, which covers the study area. The Miyagi Prefectural Cancer Registry is the oldest and most accurate population-based cancer registry in Japan.^20^ The percentage of cancer at any site, as recorded on death certificates, was 11.3% for men and 13.0% for women.^20^ Cancers were coded according to the International Classification of Diseases for Oncology, Third Edition (ICD-O-3) as esophageal cancer (C15.0–C15.9), gastric cancer (C16.0–C16.9), colorectal cancer (C18.0–C20.9), liver cancer (C22.0–C22.1), pancreatic cancer (C25.0–C25.9), malignant neoplasms of the respiratory system and intrathoracic organs (C33.0–C39.8), breast cancer (C50.0–C50.9), prostate cancer (C61.9), malignant neoplasms of urinary organs (C64.9–C68.9), all cancers (C00.0–C80.9), and non-liver cancers (C00.0–C21.8 or C23.9–C80.9).

### Variables

GGT was measured by the Szasz method under nonfasting conditions.^21^^,^^22^ The participants were divided by GGT level into 4 quartiles: GGT <13 IU/ml, 13.0 to 17.9 IU/ml, 18.0 to 29.9 IU/ml, and 30.0 IU/ml or higher. The details of the survey of cancer risk factors have been described elsewhere.^18^^,^^23^ At the baseline survey in 1994, we used a self-reported questionnaire to collect information on personal and family history of disease, smoking habits, job status, level of education, body weight, height, participation in sports or exercise, and time spent walking per day. We also asked about drinking habits, including frequency of alcohol consumption, and the quantity and type of alcoholic beverages consumed. We then classified alcohol consumption status into 4 categories: never drinkers, former drinkers, light current drinkers (<45.6 g ethanol/day on average), and heavy current drinkers (≥45.6 g ethanol/day on average). We conducted a validation study in which 113 participants provided four 3-day dietary records (including details of alcoholic beverages) within a period of 1 year and subsequently responded to the questionnaire. The Spearman’s rank correlation coefficient between the amount of alcohol consumed according to the questionnaire and the amount consumed according to the dietary records was 0.70 for men and 0.60 for women; the correlation between consumption levels measured by the 2 questionnaires administered 1 year apart was 0.76 for men and 0.66 for women.^23^

### Statistical analysis

The Cox proportional hazards regression model was used to estimate hazard ratios (HRs) and 95% confidence intervals (CIs) for cancer incidence according to GGT quartile and to adjust for potential confounding variables. SAS version 9.2 (SAS Institute, Inc., Cary, NC, USA) statistical software was used for the analysis. An HR was computed for each GGT quartile, with the lowest quartile (GGT <13 IU/ml, Q1), used as the reference group. All reported *P* values were 2-sided, and estimates with a *P* value less than 0.05 were considered statistically significant. Because the distribution of GGT was skewed to the right, a test for trend was computed using log-transformed GGT as a continuous value. All HRs were also calculated in an age- and sex-adjusted model (model 1) and in a multivariate-adjusted model (model 2). In the multivariate model, we considered the following variables to be potential confounders: age (continuous variable, years), sex, drinking habit (never drinker, former drinker, light current drinker, heavy current drinker), self-reported history of liver disease, smoking habit (never smoker, former smoker, current smoker), body mass index (<18.5 kg/m^2^, 18.5–25.0 kg/m^2^, ≥25 kg/m^2^), education (less than high school, high school or higher), time spent walking (<30 min/day, ≥30 min/day) and time spent on sports (exercise <1 hour/week, exercise ≥1 hour/week). To eliminate reverse causation, we repeated all analyses after excluding participants who suffered from cancers in the first 3 years of follow-up (model 3). The multivariate HRs and 95% CIs for non-liver cancer and for individual sites were also calculated. We also conducted stratified analyses by drinking habit (never drinker, former drinker, current drinker), smoking habit (never smoker, former smoker, current smoker), and sex. We stratified the data by drinking habit because chronic and excessive alcohol consumption increases GGT and is associated with an increased risk of some cancers, especially those of the esophagus, liver, pancreas, colorectum, and breast.^12^^–^^15^ We stratified the data by smoking habit because there was a higher prevalence of smokers in the highest GGT quartile (Table 1). We stratified the data by sex because GGT distribution in men was different from that in women. In addition, we examined the statistical significance of interaction terms, namely, the products of GGT quartile and drinking habit, GGT quartile and smoking habit, and GGT quartile and sex.

**Table 1.** Characteristics of study population by quartile of serum gamma-glutamyltransferase (GGT) level

	**Quartile of GGT (IU/L)**			

	Q1	Q2	Q3	Q4
GGT	<13	13.0–17.9	18.0–30.9	≥31.0
No. at risk	3471	3547	4240	3773

Mean GGT, IU/L	9.8	14.8	22.6	69.6
(SD)	(2.7)	(1.5)	(3.6)	(74.0)
Women (%)	85.7	70.9	50.0	27.8
Mean age, years	60.6	61.7	61.4	59.1
(SD)	(10.0)	(9.1)	(8.9)	(9.4)
Mean BMI, kg/m^2^	22.9	23.4	23.9	24.3
(SD)	(2.8)	(2.9)	(3.1)	(3.0)
Self-reported history of liver disease (%)	2.6	2.9	4.6	9.2
Current smoker (%)	10.5	17.1	27.8	43.7
Current drinker (%)	22.7	34.8	51.6	75.4
<45.6 g/day (%)	19.7	28.8	36.2	37.6
>45.6 g/day (%)	3.0	6.0	15.4	37.8
Walking ≥30 minutes/day (%)	49.7	46.7	44.6	43.8
Sports ≥1 hour/week (%)	29.3	32.3	34.8	31.9
Education (high school or more) (%)	45.6	42.4	41.1	44.6

Abbreviations: BMI, body mass index; SD, standard deviation.

## RESULTS

After a total of 130 649 person-years of follow-up, we documented 1505 cancers (947 in men, 558 in women). Of the study subjects, 9.4% were lost to follow-up. Table 1 shows the baseline characteristics of the study participants according to GGT quartile. In comparison with participants in the lowest GGT quartile (Q1), those in the highest quartile (Q4) were younger and more likely to be male, obese, a smoker, an alcohol drinker, less active (in terms of walking time), and to have liver disease. There was also a higher percentage of heavy current drinkers in the highest quartile.

Table 2 shows the HRs for any cancer and for cancers at major sites by GGT quartile. The multivariate HRs (95% CI) for any cancer were 1.03 (0.87–1.22) in Q2, 1.09 (0.92–1.28) in Q3, and 1.28 (1.08–1.53) in Q4 (*P* for trend, 0.0008 [model 2]). The multivariate HR for any cancer remained significantly higher after excluding subjects who developed cancer in the first 3 years of follow-up: 1.28 (1.06–1.55) in Q4 (*P* for trend, 0.0007 [model 3]).

**Table 2.** Hazard ratios (95% CI) for any cancer and cancers at selected sites by quartile of serum gamma-glutamyltransferase (GGT) level

	**Quartile of GGT (IU/L)**				

	**Q1**	**Q2**	**Q3**	**Q4**	
	<13	13.0–17.9	18.0–30.9	≥31.0	*P for trend*
No. at risk	3471	3547	4240	3773	
All cancers					
person-years	30 216	31 060	36 950	32 423	
No. of cases	252	314	447	492	
crude	1.00	1.21 (1.03–1.43)	1.45 (1.25–1.70)	1.82 (1.57–2.12)	
model 1	1.00	1.03 (0.87–1.22)	1.08 (0.92–1.26)	1.32 (1.12–1.56)	
model 2	1.00	1.03 (0.87–1.22)	1.09 (0.92–1.28)	1.28 (1.08–1.53)	*0.0008*
model 3	1.00	0.98 (0.81–1.18)	1.09 (0.91–1.30)	1.28 (1.06–1.55)	*0.0007*

Liver cancer					
person-years	31 015	32 071	38 230	33 772	
No. of cases	5	8	16	46	
crude	1.00	1.55 (0.51–4.73)	2.59 (0.95–7.08)	8.44 (3.35–21.24)	
model 1	1.00	1.39 (0.45–4.26)	2.23 (0.80–6.21)	8.00 (3.05–21.03)	
model 2	1.00	1.40 (0.46–4.31)	1.99 (0.72–5.56)	6.57 (2.48–17.41)	*<0.0001*
Non-liver cancer					
person-years	30 227	31 082	36 986	32 495	
No. of cases	247	306	431	451	
crude	1.00	1.21 (1.02–1.43)	1.43 (1.22–1.67)	1.70 (1.46–1.99)	
model 1	1.00	1.02 (0.86–1.21)	1.05 (0.89–1.24)	1.21 (1.02–1.44)	
model 2	1.00	1.02 (0.86–1.21)	1.06 (0.90–1.25)	1.19 (1.00–1.42)	*0.04*

Esophageal cancer					
person-years	31 008	32 073	38 242	33 764	
No. of cases	6	9	15	31	
model 2	1.00	0.93 (0.33–2.65)	0.91 (0.34–2.44)	1.53 (0.58–4.03)	*0.2*
Stomach cancer					
person-years	30 812	31 794	37 971	33 533	
No. of cases	55	88	99	100	
model 2	1.00	1.26 (0.89–1.77)	0.96 (0.68–1.36)	1.00 (0.69–1.45)	*0.6*
Respiratory/intrathoracic cancer					
person-years	30 977	32 010	38 073	33 733	
No. of cases	31	45	79	61	
model 2	1.00	1.10 (0.69–1.74)	1.36 (0.88–2.10)	1.13 (0.69–1.82)	*0.4*
Pancreatic cancer					
person-year	31 014	32 088	38 241	33 838	
No. of cases	10	12	25	20	
model 2	1.00	1.08 (0.46–2.51)	1.81 (0.84–3.90)	1.89 (0.81–4.38)	*0.2*
Colorectal cancer					
person-years	30 859	31 823	37 949	33 427	
No. of cases	45	58	83	114	
model 2	1.00	1.07 (0.73–1.59)	1.12 (0.77–1.64)	1.57 (1.06–2.32)	*0.02*
Kidney and urinary cancer					
person-years	30 977	32 022	38 178	33 779	
No. of cases	14	21	28	20	
model 2	1.00	1.29 (0.65–2.55)	1.34 (0.69–2.63)	1.10 (0.51–2.36)	*0.7*
Prostate cancer					
No. at risk	497	1031	2119	2725	
person-years	4327	9114	18 949	24 399	
No. of cases	18	32	55	45	
model 2	1.00	0.95 (0.53–1.70)	0.92 (0.53–1.58)	0.75 (0.42–1.34)	*0.1*
Breast cancer					
No. at risk	2974	2516	2121	1048	
person-years	26 547	22 785	19 096	9300	
No. of cases	24	20	16	11	
model 2	1.00	1.03 (0.56–1.87)	0.98 (0.52–1.87)	1.39 (0.67–2.92)	*0.8*

model 1: adjusted for sex and age (continuous variable, years).

model 2: model 1 + alcohol consumption (never, former, currently <45.6 g/day, currently >45.6 g/day ethanol), self-reported history of liver disease, cigarette smoking (never, former, current), body mass index (<18.5, 18.5 to <25.0, ≥25.0 kg/m^2^), education (junior high school, high school or more), walking (<30 minutes/day, >30 minutes/day) and sports (rarely, >1 hour/week).

model 3: excluded participants who developed cancers in the first 3 years of follow-up in model 2.

*P* for trend was computed with log-transformed GGT as a continuous value in the multivariate model.

Because of the clinically obvious positive association between GGT and liver cancer, we repeatedly examined the association of GGT level with liver cancer and non-liver cancer. The multivariate HR (95% CI) in Q4 for liver cancer was 6.57 (2.48–17.41). That for non-liver cancers was attenuated to 1.19 (1.00–1.42) but was nevertheless significantly elevated. Regarding the analysis of selected cancer sites, the multivariate HR for colorectal cancer was significantly elevated: 1.57 (1.06–2.32; *P* for trend, 0.02). The HRs for esophageal, pancreatic, and breast cancer were also elevated (1.53, 1.89, and 1.39, respectively) but not significantly so. All these cancers (colorectal, esophageal, pancreatic, and breast cancers) are alcohol-related.^12^^–^^15^ In contrast, the risks of other, non–alcohol-related cancers, ie, stomach, respiratory and intrathoracic, kidney and urinary, and prostate cancers, were not increased.

### Stratified analysis

We examined the multivariate HRs for non-liver cancers using stratified analysis (Table 3). When stratified by drinking habit, the multivariate HRs (95% CI) for non-liver cancers in Q4 were 1.03 (0.74–1.43) in never drinkers and 1.30 (0.96–1.75) in current drinkers. A significant trend was observed in current drinkers but not in never drinkers. When stratified by smoking habit, the multivariate HRs for non-liver cancers in Q4 were 1.10 (0.82–1.46) in never smokers and 1.63 (1.12–2.37) in current smokers. A significant trend was observed in current smokers but not in never smokers. When stratified, the multivariate HR was 1.16 in men and 1.22 in women. Thus, there was no apparent sex difference in the association between GGT level and cancer incidence, even though the distribution of GGT differs between men and women. We added the various interaction terms to our multivariate model, ie, the products of GGT quartile and drinking habit, GGT quartile and smoking habit, and GGT quartile and sex. However, none of these interaction terms were statistically significant: the interaction *P* values were 0.19, 0.43, and 0.44, respectively.

**Table 3.** Hazard ratios (HRs) and 95% CIs for incidence of non-liver cancer by quartile of serum gamma-glutamyltransferase (GGT) level in stratified analysis

	**Quartile of GGT (IU/L)**				*P for trend*

	**Q1**	**Q2**	**Q3**	**Q4**	
Never drinkers					
person-years	17 983	16 064	13 959	6288	
No. of cases	124	138	148	52	
HR^a^	1.00	1.09 (0.85–1.40)	1.24 (0.97–1.59)	1.03 (0.74–1.43)	*0.2*
Former drinkers					
person-years	1216	1421	1874	1177	
No. of cases	29	18	31	22	
HR^a^	1.00	0.47 (0.26–0.85)	0.70 (0.41–1.18)	0.76 (0.42–1.37)	*0.54*
Current drinkers					
person-years	5648	9202	17 016	23 058	
No. of cases	55	111	217	358	
HR	1.00	1.04 (0.75–1.44)	1.03 (0.77–1.40)	1.30 (0.96–1.75)	*0.02*

Never smokers					
person-years	20 420	18 195	17 680	10 344	
No. of cases	140	140	141	89	
HR^b^	1.00	1.05 (0.83–1.33)	1.03 (0.81–1.32)	1.10 (0.82–1.46)	*0.58*
Former smokers					
person-years	1328	2961	5675	6447	
No. of cases	29	52	88	94	
HR^b^	1.00	0.78 (0.49–1.23)	0.73 (0.47–1.11)	0.76 (0.49–1.18)	*0.66*
Current smokers					
person-years	2474	4252	8617	12 917	
No. of cases	37	71	157	228	
HR^b^	1.00	1.17 (0.79–1.75)	1.41 (0.98–2.04)	1.63 (1.12–2.37)	*0.03*

Men					
No. at risk					
person-years	4164	8796	18 288	23 400	
No. of cases	83	152	285	380	
HR^c^	1.00	0.97 (0.74–1.27)	0.96 (0.75–1.24)	1.16 (0.90–1.50)	*0.04*
Women					
No. at risk					
person-years	26 063	22 286	18 698	9095	
No. of cases	164	154	146	71	
HR^c^	1.00	1.06 (0.85–1.32)	1.20 (0.96–1.50)	1.22 (0.92–1.62)	*0.1*

Each multivariate HR was adjusted for sex, age (continuous variable, years), alcohol consumption (never, former, currently drinking <45.6 g/day, currently drinking >45.6 g/day ethanol), self-reported history of liver disease, cigarette smoking (never, former, currently smoking), body mass index (<18.5, 18.5 to <25.0, or ≥25.0 kg/m^2^), education (junior high school, high school or more), walking (<30 minutes/day, >30 minutes/day) and sports (rarely, >1 hour/week).

^a^Alcohol consumption was not considered in this stratified model.

^b^Cigarette smoking was not considered in this stratified model.

^c^Sex was not considered in this stratified model.

*P* for trend was computed with log-transformed GGT as a continuous value in the multivariate model.

## DISCUSSION

In this prospective population-based cohort of adults living in Japan, we found a significant relationship between GGT level and cancer incidence—after adjusting for a number of confounders—during a follow-up period of 10 years. A positive trend was observed in current drinkers (*P* for trend = 0.02) but not in never drinkers.

Experimental data show that GGT level reflects the degree of oxidative stress, a key factor in tumor progression.^6^^–^^9^ Thus, it could be hypothesized that the association between GGT and carcinogenesis is not specific to liver cancer but rather is generalizable to all cancers. However, the present results do not support this hypothesis.

The only published epidemiologic study on the association of GGT with cancer incidence, which was conducted in Austria, reported a positive association of GGT level with cancer risk.^10^^,^^11^ That study documented an increased risk of cancer in digestive organs, respiratory system/intrathoracic organs, urinary organs (in men), breast and female genital organs (in women), and lymphoid and hematopoietic cancers (in women). Our present results agree with those findings in that GGT was positively associated with cancers of the digestive organs and breast. The discrepancy regarding cancers of the respiratory system/intrathoracic organs could have been due to a difference in the definition of “respiratory system/intrathoracic organs”, as neoplasms of the nasal cavity and pharynx were included in the Austrian study but not in ours. When we examined the multivariate HR for neoplasms of respiratory system/intrathoracic organs, including the nasal cavity and pharynx, the HR was 1.22 (0.77–1.96), ie, slightly elevated. We were unable to fully evaluate the association of GGT with hematologic malignancies because of the limited number of such malignancies in our cohort (23 cases in total).

Positive associations of GGT with cancer incidence have been observed for the esophagus, liver, pancreas, colorectum, and breast, ie, cancers for which alcohol drinking is known to increase risk.^12^^–^^15^ The fact that a positive association was observed only for these alcohol-related cancers indicates that the associations were attributable to residual confounding by alcohol. Furthermore, the positive association was observed only in current drinkers, among whom residual confounding by alcohol is plausible. This further supports our hypothesis that the positive associations were due to residual confounding by alcohol.

Unexpectedly, a positive association of GGT with cancer incidence was observed in current smokers but not in never smokers (Table 3). The increased HR remained significant even after further adjustment for pack-years smoked (packs per day × years smoked; data not shown). This increment might be due the fact that the HR for esophageal cancer in Q4 tended to be higher (1.72 [0.46–6.44]), whereas those for other alcohol-related cancers did not. In addition, the increment may also be due to alcohol consumption. When we examined participants who currently smoked and drank (*n* = 2483), the multivariate HR in Q4 was 1.87 (1.09–3.20). By contrast, when we examined participants who currently drank and never smoked (*n* = 519), the multivariate HR in Q4 was 1.05 (0.41–2.70). These results suggest that the increased HR in Q4 among current smokers largely reflected the effect of alcohol consumption.

### Strengths and limitations

We investigated the effect of alcohol consumption on the association between GGT level and cancer incidence. Our questionnaire on alcohol consumption was well validated. We conducted a validation study in which 113 participants provided four 3-day dietary records. The Spearman correlation coefficient between the amount of alcohol consumed according to the questionnaire and the amount consumed according to the dietary records was 0.70 for men and 0.60 for women; the correlation between consumption measured by the 2 questionnaires administered 1 year apart was 0.76 for men and 0.66 for women.^23^

This study did have some limitations. First, we used part of a cohort, ie, those who attended an annual health checkup in 1995. However, we compared cancer incidence between those who did and did not attend this health checkup and found that the rate did not differ between groups: the multivariate HR (95% CI) for any cancer in subjects who attended the 1995 checkup was 0.98 (0.92–1.04) relative to those who did not. This suggests that the present results are generalizable to our original population. A second limitation is that information on whether participants had liver disease was determined from a self-reported questionnaire. We did not collect any information on hepatitis B/C virus infection, nor did we perform abdominal ultrasonography. However, these factors would affect only liver cancer and not non-liver cancers. Finally, 9.4% of the total participants were lost to follow-up. This proportion did not vary largely across the GGT quartiles; the proportions were 11.3%, 9.6%, 8.8%, and 8.1% of participants in the lowest to the highest GGT quartiles, respectively. Therefore, we consider it unlikely that the association between GGT and cancer incidence was substantially distorted because of loss to follow-up.

Recent experimental evidence has indicated that GGT is a sensitive and reliable marker of oxidative stress,^1^^–^^5^ a key factor involved in tumor pathogenesis via glutathione metabolism.^7^^–^^9^ On the basis of experimental studies, a general causative of GGT with carcinogenesis has been reported, but associations have not been reported for specific sites. However, the present results do not support the hypothesis that GGT is related to cancer in general. A positive association was observed only for specific cancers that are related to alcohol consumption. In addition, this positive association was observed only in current drinkers.

In conclusion, our results suggest that the positive association of GGT with cancer incidence largely reflects alcohol consumption. Our findings confirm the importance of considering alcohol consumption when attempting to interpret the association of GGT with cancers and show that GGT could be a marker of alcohol-related cancers, which indicates that individuals who a have high level of GGT should be assessed for alcohol-related cancers, such as those of the esophagus, liver, pancreas, colorectum, liver, and breast.
